# AUNIP was a candidate marker for prognosis and immunology in pan-cancer

**DOI:** 10.1007/s13205-025-04294-6

**Published:** 2025-05-17

**Authors:** Xiaorong Guo, Ting Liu, Nan Li, Li Jin

**Affiliations:** 1 Department of Pathology, The Second Affiliated Hospital of Harbin Medical University, Harbin, 150086 Heilongjiang China; 2 Department of Pathology, Beijing Ditan Hospital, Capital Medical University, No. 8 Jing Shun East Street, Chaoyang District, Beijing, 100015 China; 3 Department of Pathology, The Fourth Affiliated Hospital of Harbin Medical University, Harbin, 150081 Heilongjiang China; 4 Cancer Center, Department of Pathology, Zhejiang Provincial People’s Hospital, Affiliated People’s Hospital, Hangzhou Medical College, Hangzhou, Zhejiang China

**Keywords:** AUNIP, Pan-cancer, Prognostic, Immunity, Hepatocellular carcinoma

## Abstract

AUNIP (Aurora kinase A[Aurora-A] and ninein-interacting protein), is a key factor regulating the end-state of DNA cleavage. It has been reported that AUNIP affects the progression of some tumors; however, the molecular functions involved in AUNIP remain unknown. We employed some databases, such as TCGA, GTEx, TIMER, GEPIA2, cBioportal, and GSCALite, to study AUNIP gene expression, prognosis, gene variation, and drug sensitivity. The relationship between AUNIP and clinicopathological information was explored using Wilcoxon test. The association between AUNIP and TMB, MSI, immunocyte infiltration, and immune checkpoints were analyzed using Spearman correlation analysis. We employed GSEA to research the functional mechanisms involved in AUNIP for pan-cancer. Moreover, we conducted immunohistochemistry (IHC) to investigate AUNIP difference expression between liver hepatocellular carcinoma (LIHC) and normal tissues. The Chisq test was used to study the correlation of AUNIP with clinical characteristics. AUNIP was highly expressed in majority of tumors and IHC analysis demonstrated that AUNIP expression was higher in LIHC than normal tissues. AUNIP overexpression had adverse outcomes in adrenocortical carcinoma (ACC), brain lower grade glioma (LGG), LIHC, mesothelioma (MESO), and sarcoma (SARC). Furthermore, high AUNIP expression led to unfavorable prognosis in LIHC. AUNIP was associated with T stage, N stage, and clinicopathological analysis in several cancers and AUNIP expression had a correlation with histologic grade in LIHC by IHC. Mutation analysis showed that AUNIP was the highest frequency of genetic changes in cholangiocarcinoma (CHOL). AUNIP was negatively associated with 30 small-molecule drugs that inhibit tumor development. AUNIP expression had association with TMB, MSI, immune cell infiltration, and immune checkpoints for various tumors. GSEA results suggested that AUNIP mainly participated in the cell cycle, DNA replication, mismatch repair, and homologous recombination.Pan-cancer study considered AUNIP as a potential prognostic marker and high latent diagnostic biomarker.

**Electronic supplementary material:**

The online version of this article (10.1007/s13205-025-04294-6) contains supplementary material, which is available to authorized users.

## Introduction

Cancer is a serious disease that becomes a threat to mankind's health. The number of deaths from cancer is increasing every year. Pan-cancer research has been prevalent in recent years, with the aim of integrating TCGA data based on different tumor types and platforms, while analyzing and interpreting these data. Our research relies on multi-omics database to explore differences between tumors, guiding tumor diagnosis, prognosis, and treatment selection (Zhang and Wang 2015; Yang et al. 2018).

AUNIP (Aurora kinase A and Ninein-interacting protein) is a centrosomal protein that interacts to promote the maintenance of Aurora-A and Ninein centrosome structures and the formation of spindles (Zhang and Wang 2015). AUNIP regulates the mitotic entry and mitotic spindle assembly by activating of Plk1 and Aurora-A. Yang et al. used bioinformatics to investigate the high expression of AUNIP in oral squamous cell carcinoma (OSCC), which is associated with tumor microenvironment, human papillomavirus infection, and cell cycle. Inhibition of AUNIP can inhibit OSCC cells’ proliferation, resulting in the G0/G1 phase arrest of OSCC cells. AUNIP overexpression predicts bad prognosis in OSCC patients (Yang et al. 2019). However, there are few reports on pan-cancer research in AUNIP.

Our work used bioinformatics aspect to discuss the expression, prognosis, clinicopathological features, mutation, tumor mutation load (TMB), microsatellite instability (MSI), immune characteristics, and drug sensitivity of AUNIP from the viewpoint of pan-cancer, and comprehensively analyzed the characteristics and mechanism of AUNIP, providing new ideas for tumor treatment and prognosis.

## Materials and methods

### Differential expression of AUNIP mRNA for cancers and normal samples

TIMER2 database studied immune cells infiltration in different tumors, as well as the differential expression of 33 kinds of tumors and normal tissues from TCGA database (Li et al. 2020). Owing to the absence of normal samples in several tumors in TIMER database, we merged TCGA and GTEx to discuss the differential expression of AUNIP in 33 tumors and normal tissues.

### Prognosis and diagnostic value of AUNIP

GEPIA2 database is an online platform in which survival significance maps of genes in pan-cancer can be obtained. According to the median value of AUNIP expression, AUNIP was divided into low-expression group and high-expression group and Kaplan–Meier was used to show prognostic differences of both groups (Tang et al. 2019). In addition, a receiver-operating characteristic (ROC) curve estimated the diagnose value for AUNIP using pROC in R.

### Clinicopathological features

We acquired the expression data and clinicopathological parameters of 33 cancers from the TCGA database and utilized Wilcoxon test to investigate its correlation with clinicopathology, including T stage, N stage, and pathological stage.

### Immunohistochemical staining

Forty-four cases of liver cancer and paracancerous tissue were collected from the Department of Pathology of the Zhejiang Provincial People’s Hospital. The study was authorized by the ethics committee of Zhejiang Provincial People’s Hospital (batch number: QT2025083), and all patients received written informed consent before surgery. Patients with a pathologic diagnosis of hepatocellular carcinoma who had not received any preoperative chemotherapy or radiotherapy were included in the study. Patients with other diagnosed malignancies were not included in the study. The slices were cut into 4 um thick. The sections were dewaxed, hydrated, and repaired by high-pressure antigen. The endogenous catalase activity was inactivated by 3% H_2_O_2_ at room temperature for 10 min. The non-specific antigen was blocked by 10% sheep serum after rinsed with PBS at 37 ℃ for 10 min, and rabbit anti-AUNIP polyclonal antibody (bs-15019R, 1:200, Bioss Company) was added at 4 ℃ overnight. The next day, the secondary antibody (Goat anti-Rabbit IgG, PV-6000, Beijing Zhongshan Jinqiao Biotechnology Co., Ltd.) was added, and then developed color with DAB. Finally, the slices were observed in the microscope. The standard of expression strength is: 0 points without staining; light yellow is 1 point; light brown is 2 points; dark brown is 3 points. The scoring criteria for positive cells were: 0 points for ≤ 5%; 6% ~ 25% is 1 score; 26% ~ 50% is 2 points; 51% ~ 75% is 3 points; > 76% is 4 points. AUNIP expression is interpreted by the percentage of positive cells multiplied by the staining intensity. The degree of positive staining was defined: ≤ 7 is classified as low expression, and > 7 is classified as high expression.

### Mutational analysis of AUNIP

cBioPortal studied the frequency of AUNIP gene change in various tumors (Cerami et al. 2012).

### Correlative analysis of AUNIP expression with TMB and MSI

TMB is the total number of genetic coding errors, base substitution, gene insertion, or deletion errors detected in somatic cells from millions of bases, and it can effectively evaluate tumor mutation and neoantigen load and is related to immunotherapy response (Zhang et al. 2019; Chan et al. 2019). MSI is due to mismatch repair gene defects. Tumors with MSl molecular characteristics increase tumor antigen load due to high-frequency gene mutation, inducing killer T lymphocyte infiltration and corresponding immunosuppressive molecule high expression, and respond well to corresponding immunotherapy (Dudley and Le 2016). The interrelation of AUNIP expression with TMB and MSI in 33 tumors was discussed by Spearman analysis.

### Association of AUNIP with immune cell infiltration and immune checkpoints

Cells and molecules of the tumor microenvironment (TME) can influence the efficiency of immunotherapy, so research on TME is of great significance in immunotherapy. Tumor immune cell infiltration is closely linked to tumor progression in the TME. The relationship of AUNIP with 23 types of immune cell infiltration in different cancers was applied using ssGSEA algorithm in R language. In addition, the stromal score, immune score, and estimate score for different tumors were investigated using ESTIMATE algorithm. Immunotherapy with immune checkpoint inhibitors has initiated a new era of tumor treatment, and finding predictable biomarkers is a necessary pathway for achieving precise tumor immunotherapy. At present, the eight commonest immune checkpoints are PD-1, PD-L1, CTLA-4, PDCD1LG2, TIGIT, HAVCR2, SIGLEC15, and LAG3. We discussed the association of AUNIP with immune checkpoints through Spearman analysis.

### Correlative analysis between AUNIP expression and drug sensitivity

GSCALite is an integrated platform for genomic, pharmacogenomic, and immunogenomic gene set cancer analysis. The CTRP dataset from the GSCALite database (http://bioinfo.life.hust.edu.cn/web/GSCALite/) was employed to explore the relationship between gene expression and drug sensitivity (Liu et al. 2022).

### Gene set enrichment analysis of AUNIP

We conducted GSEA analysis between high AUNIP expression and low AUNIP expression according to the KEGG dataset in the MSigDB database.

### Statistical analysis

Wilcoxon test was employed to study the difference expression between tumors and normal tissues. The relationship of AUNIP expression with clinicopathological features was applied by Chi-square test. We investigated the association of AUNIP with TME and immune checkpoints using Spearman correlation. P < 0.05 was considered statistically significant.

## Results

### Overexpression of AUNIP mRNA in various tumors

**Fig. 1:**
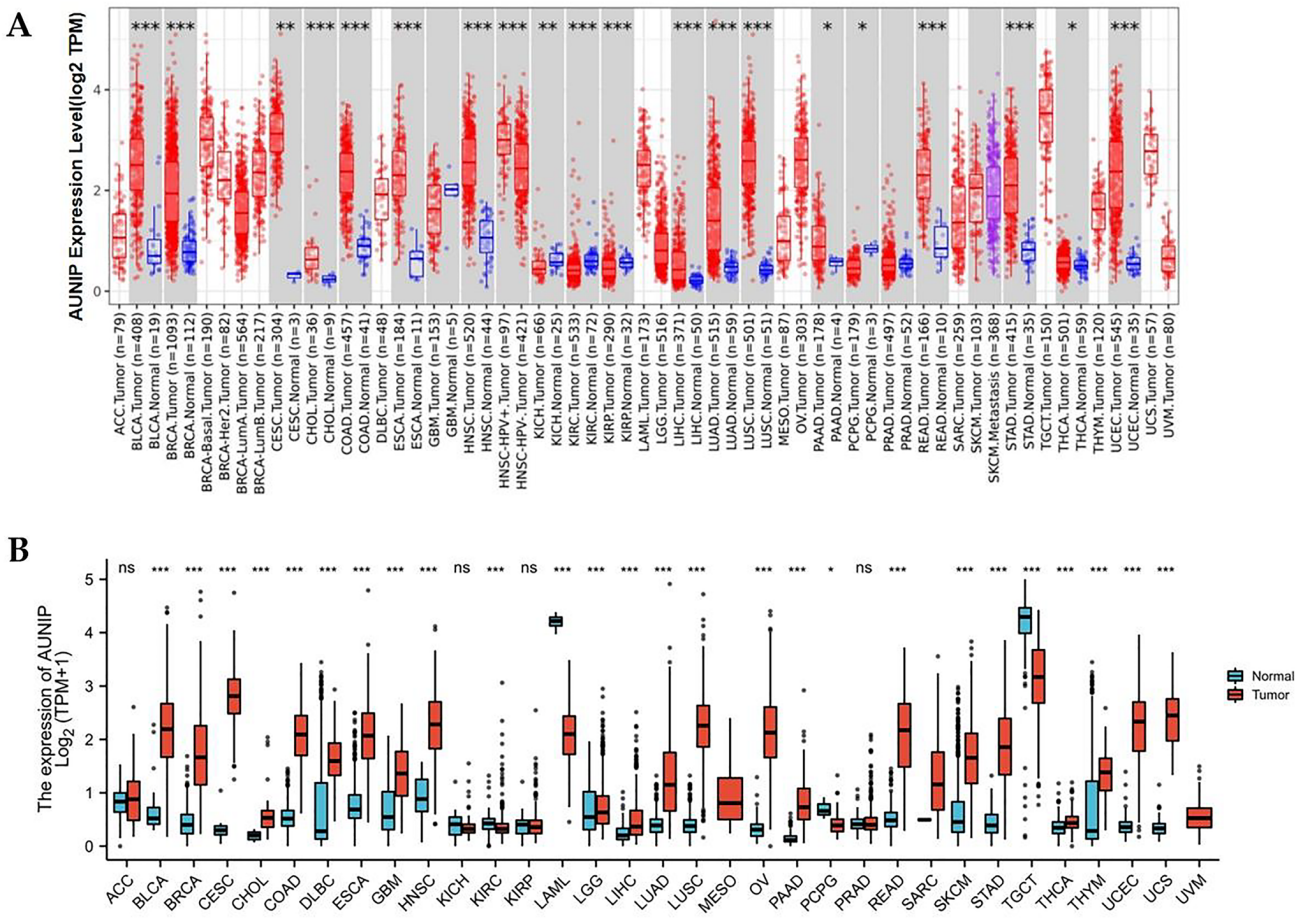
The expression of AUNIP in pan-cancer and normal tissues in TIMER database (**A**) and TCGA + GTEx (**B**) (***P < 0.001, **P < 0.01, *P < 0.05)

The TIMER database showed that compared with normal tissue, AUNIP had high expression in BLCA, BRCA, CESC, CHOL, COAD, ESCA, HNSC, LIHC, LUAD, LUSC, PAAD, READ, STAD, THCA, and UCEC, and low expression in KICH, KIRC, KIRP, and PCPG (Fig. 1A). Because of no normal tissues in some tumors in the TIMER database, TCGA was combined with GTEx to explore AUNIP expression in tumors and corresponding normal samples. We discovered that AUNIP expression in BLCA, BRCA, CESC, CHOL, COAD, DLBC, ESCA, GBM, HNSC, LGG, LIHC, LUAD, LUSC, OV, PAAD, READ, SKCM, STAD, THCA, THYM, UCEC, and UCS was higher than in normal tissues, but lower in KIRC, LAML, PCPG, and TGCT (Fig. 1B).

### Prognosis and diagnostic value of AUNIP in pan-cancer

**Fig. 2:**
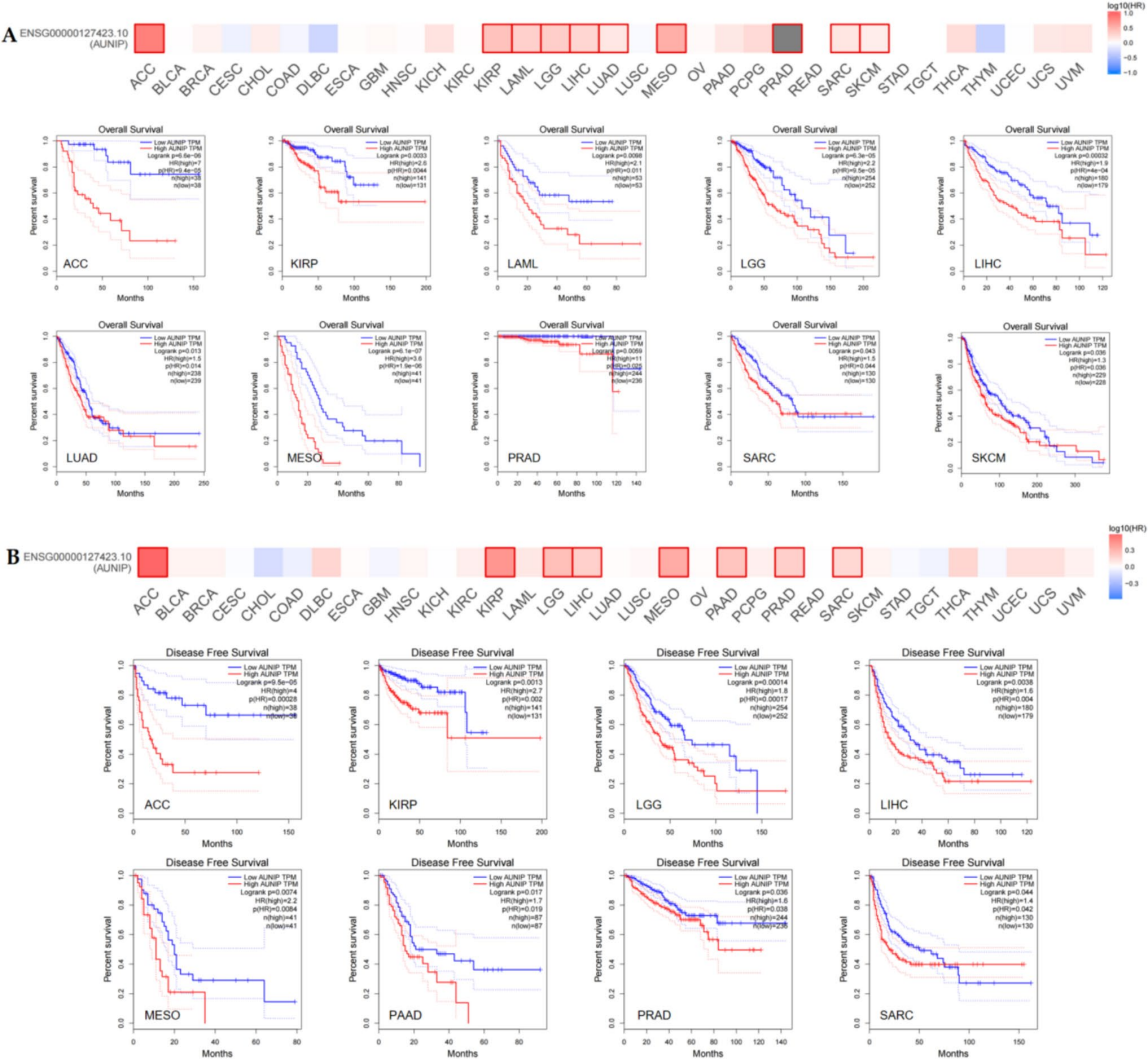
The survival hotmaps and Kaplan–Meier survival curve in pan-cancer using GEPIA2. **A** The OS of AUNIP in pan-cancer. **B** The DFS of AUNIP in pan-cancer

We analyzed the impact of AUNIP expression on survival in 33 tumors. Prognostic index included overall survival (OS) and disease-free survival (DFS). The findings demonstrated that the OS of low-expression AUNIP was better than that of high-expression AUNIP for ACC, KIRP, LAML, LGG, LIHC, LUAD, MESO, PRAD, SARC, and SKCM, (Fig. 2A). Regarding DFS, the DFS for low-expression AUNIP in ACC, KIRP, LGG, LIHC, MESO, PAAD, PRAD, and SARC was better than that of high-expression AUNIP (Fig. 2B). Among them, the OS and DFS with low AUNIP expression for ACC, KIRP, LGG, LIHC, MESO, PRAD, and SARC were higher than those with high AUNIP expression. There was no significant difference of AUNIP expression in KIRP and PRAD, compared to their corresponding normal tissues. Therefore, AUNIP was related to OS and DFS in ACC, LGG, LIHC, MESO, and SARC. Furthermore, we evaluated the diagnostic value of AUNIP in tumors. The area under the receiver-operating characteristic curve (AUC) > 0.7 is considered certain accuracy, and AUC > 0.9 is considered higher accuracy (Mishra et al. 2023a). The results demonstrated that AUNIP has high diagnostic value in BLCA, BRCA, CESC, CHOL, COAD, ESCA, HNSC, LUAD, LUSC, PCPG, READ, STAD, UCEC, and certain diagnostic values for KICH, KIRC, LIHC, PAAD, and SARC (Figure S1).

### AUNIP was associated with clinicopathological features in some tumors

**Fig. 3:**
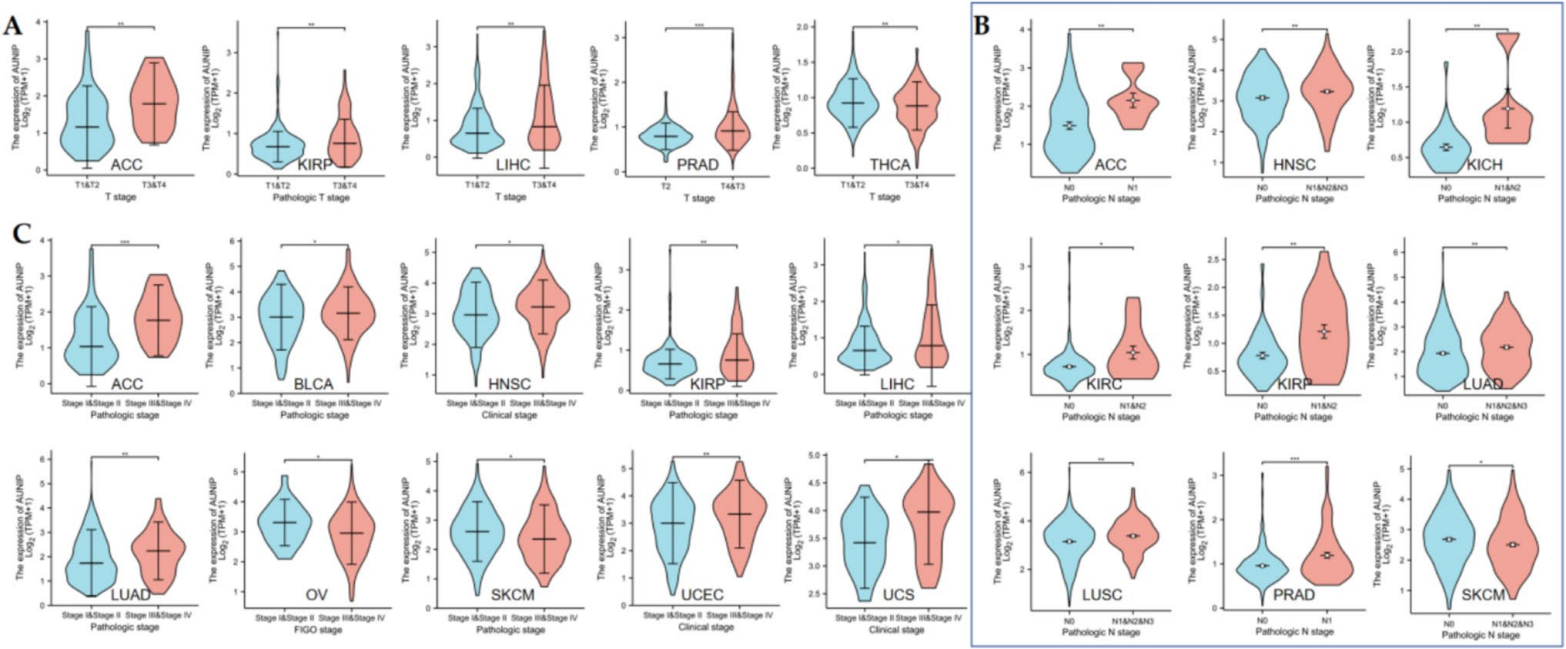
The correlation of AUNIP with clinical pathological features in some tumors. **A** The correlation of AUNIP with T stage in ACC, KIRP, LIHC, PRAD, and THCA. **B** The association between AUNIP and N stage in ACC, HNSC, KICH, KIRC, KIRP, LUAD, LUSC, PRAD, and SKCM. **C** The relationship of AUNIP with pathological stage in ACC, BLCA, HNSC, KIRP, LIHC, LUAD, OV, SKCM, UCEC, and UCS (***P < 0.001, **P < 0.01, *P < 0.05)

We downloaded clinicopathological data for 33 tumors. Regarding T staging, AUNIP expression was higher at T3 + T4 than at T1 + T2 in ACC, KIRP, LIHC, PRAD, and lower at T3+T4 in THCA (Fig. 3A). In ACC, HNSC, KICH, KIRC, KIRP, LUAD, LUSC, and PRAD, AUNIP was higher expressed in patients with N1&N2&N3 than in patients with N0, while in SKCM, AUNIP was higher expressed in patients with N0(Fig. 3B). In ACC, BLCA, HNSC, KIRP, LIHC, LUAD, UCEC, and UCS, AUNIP expression increased with the increase of pathological staging, while in OV and SKCM, AUNIP expression decreased with the increase of pathological staging (Fig. 3C).

### Gene variation of AUNIP in pan-cancer

**Fig. 4:**
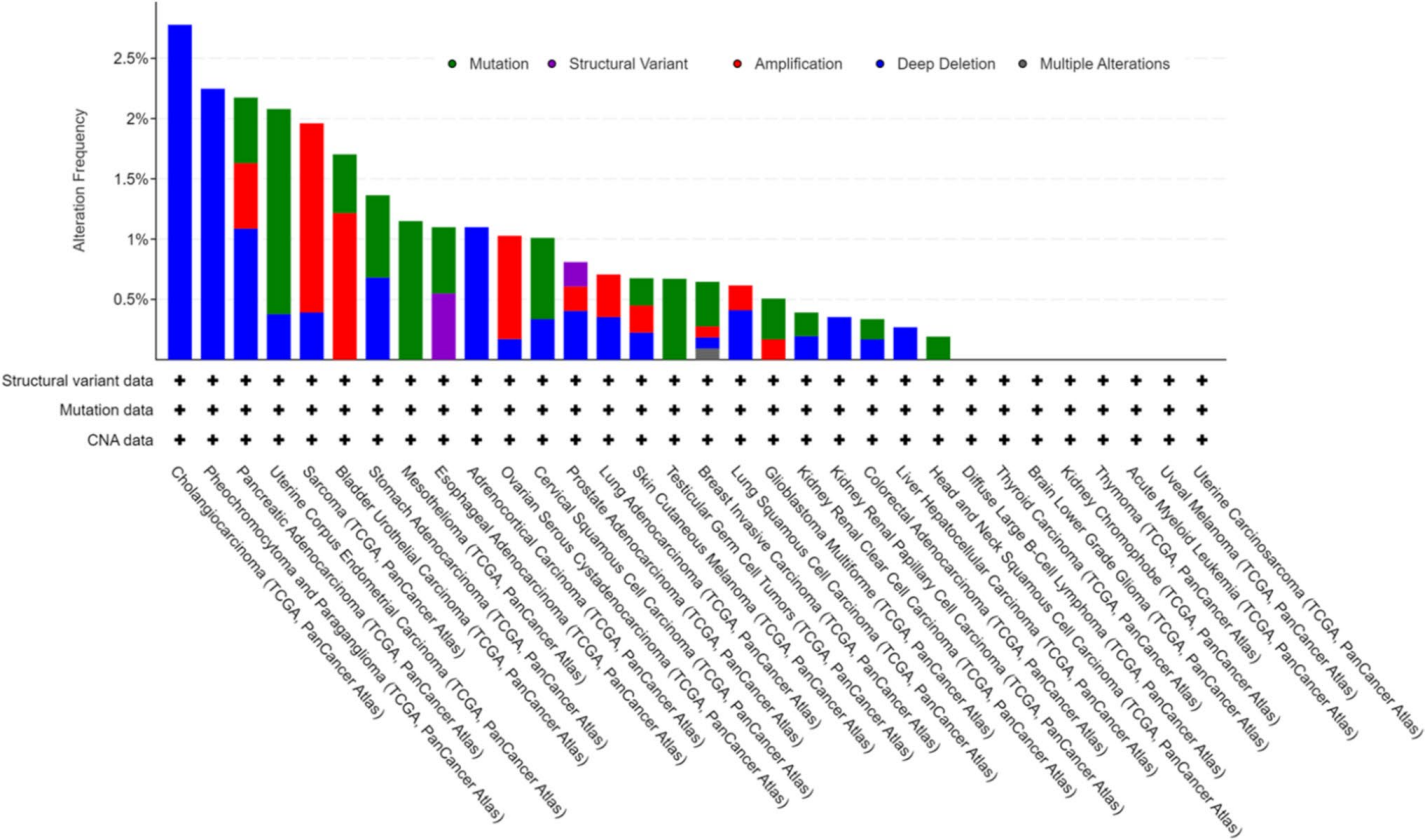
The gene alteration of AUNIP in pan-cancer

Genetic alterations are a form of epigenetics. Genetic alterations of AUNIP in different tumors appear in the form of mutation, structural variant, amplification, deep deletion, and multiple alterations. Among them, CHOL has the highest frequency of gene change, which is manifested as deep deletion, followed by PCPG, which is mainly manifested as deep deletion. The third genetic alteration is PAAD (mutation, amplification and deep deletion). No genetic alteration of AUNIP was observed in DLBC, THCA, LGG, KICH, THYM, LAML, UVM, and UCS (Fig. 4).

### AUNIP expression was related to TMB and MSI in some cancers

**Fig. 5:**
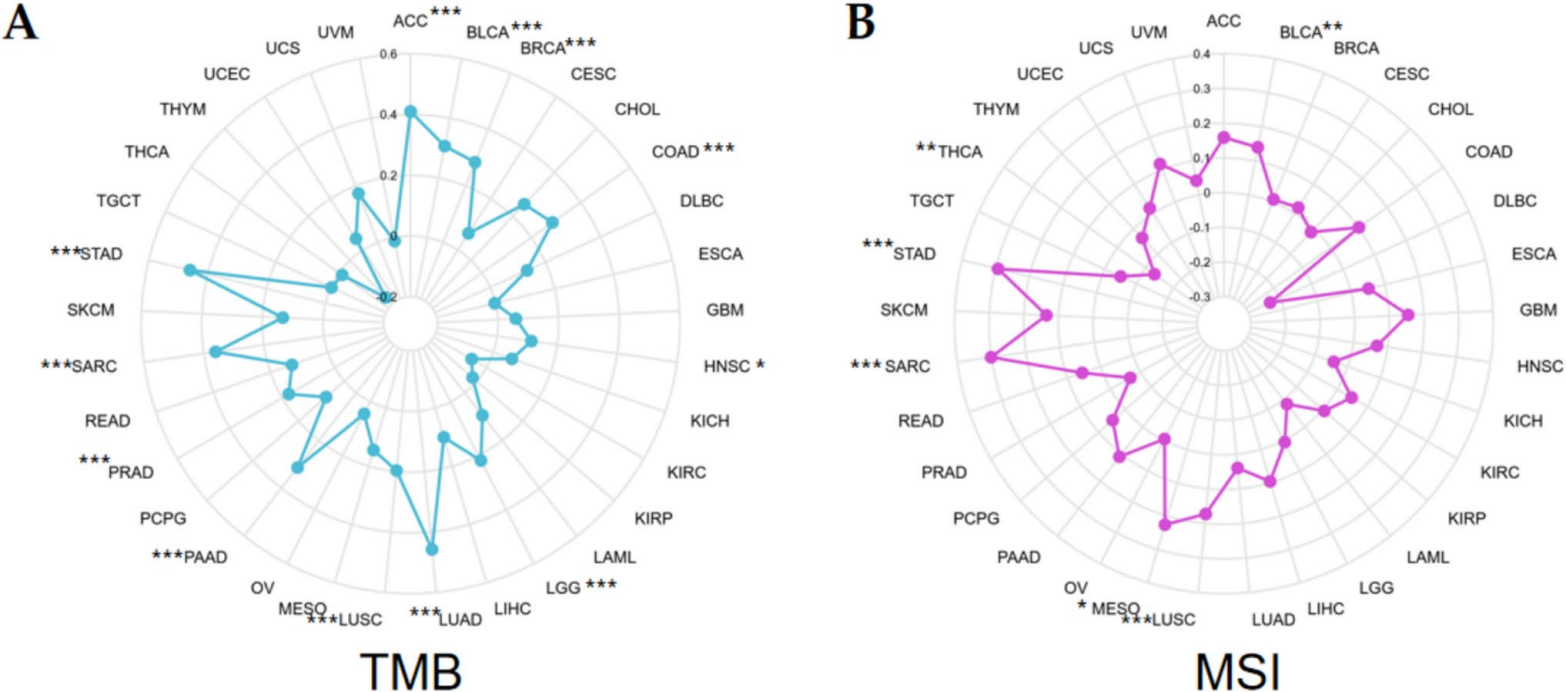
The relationship of AUNIP with TMB (**A**) and MSI (**B**) (***P < 0.001, **P < 0.01, *P < 0.05)

TMB and MSI are two common potential indicators for tumor immunotherapy response. The findings suggested that AUNIP expression had positive correlation with TMB in ACC, BLCA, BRCA, COAD, HNSC, LGG, LUAD, LUSC, PAAD, PRAD, SARC, and STAD, and the correlation coefficient with STAD is the highest (Fig. 5A). AUNIP had positive interrelation with MSI in BLCA, LUSC, MESO, SARC, and STAD, and negative association with MSI in THCA (Fig. 5B).

### Association of AUNIP with immune cell infiltration and immune checkpoints in pan-cancer

**Fig. 6:**
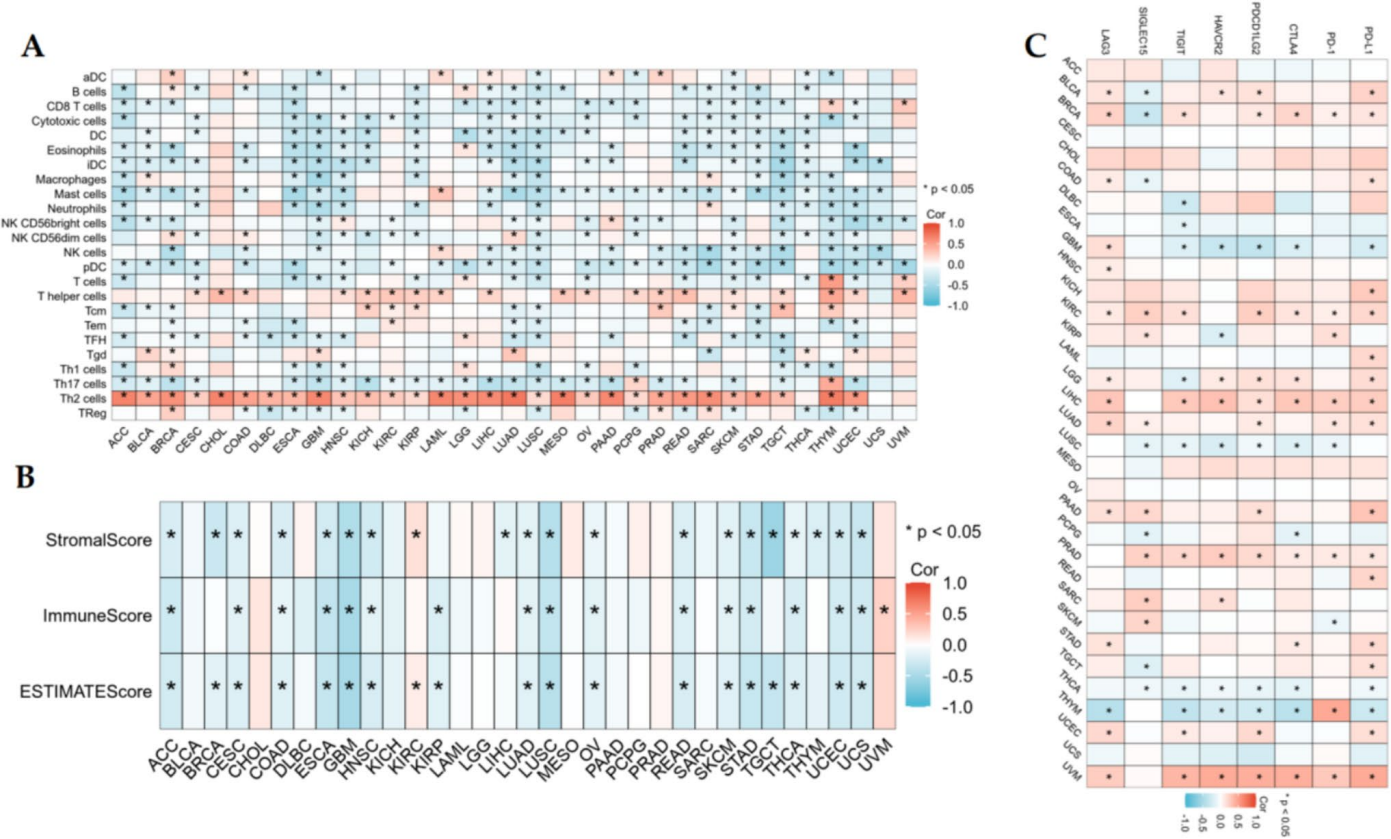
The relationship between AUNIP and immunity in pan-cancer. **A** The relationship of AUNIP with immune cell infiltration using ssGSEA. **B** The association between AUNIP and stromalscore, immunescore, and estimatescore using ESTIMATE. **C** The correlation between AUNIP and immune checkpoints. (*P < 0.05)

We applied ssGSEA to investigate the interrelation between AUNIP and 23 kinds of immune cell infiltration in pan-cancer and found that AUNIP was positively linked with Th2 cells in 30 kinds of tumors, and the positive correlation coefficient was the highest. Among the remaining immune cells, AUNIP had a correlation with one or more immune cells in different cancers (Fig. 6A). For the ESTIMATE algorithm, in ACC, CESC, COAD, ESCA, GBM, HNSC, LUAD, LUSC, OV, READ, SKCM, STAD, THCA, UCEC, and UCS, AUNIP expression had negative association with stromalscore, immunescore, and estimatescore. In BLCA, CHOL, DLBC, KICH, LAML, LGG, MESO, PAAD, PCPG, PRAD, and SARC, AUNIP was not associated with stromalscore, immune score, and estimatescore, and AUNIP was related to stromalscore, immunescore, and estimate score in the remaining 7 tumors(Fig. 6B). Immune checkpoint is the target of immunotherapy at this stage, and our results showed that AUNIP was positively or negatively associated with an immune checkpoint in all tumors except ACC, CESC, CHOL, MESO, OV, and UCS (Fig. 6C).

### Drug sensitivity analysis

**Fig. 7:**
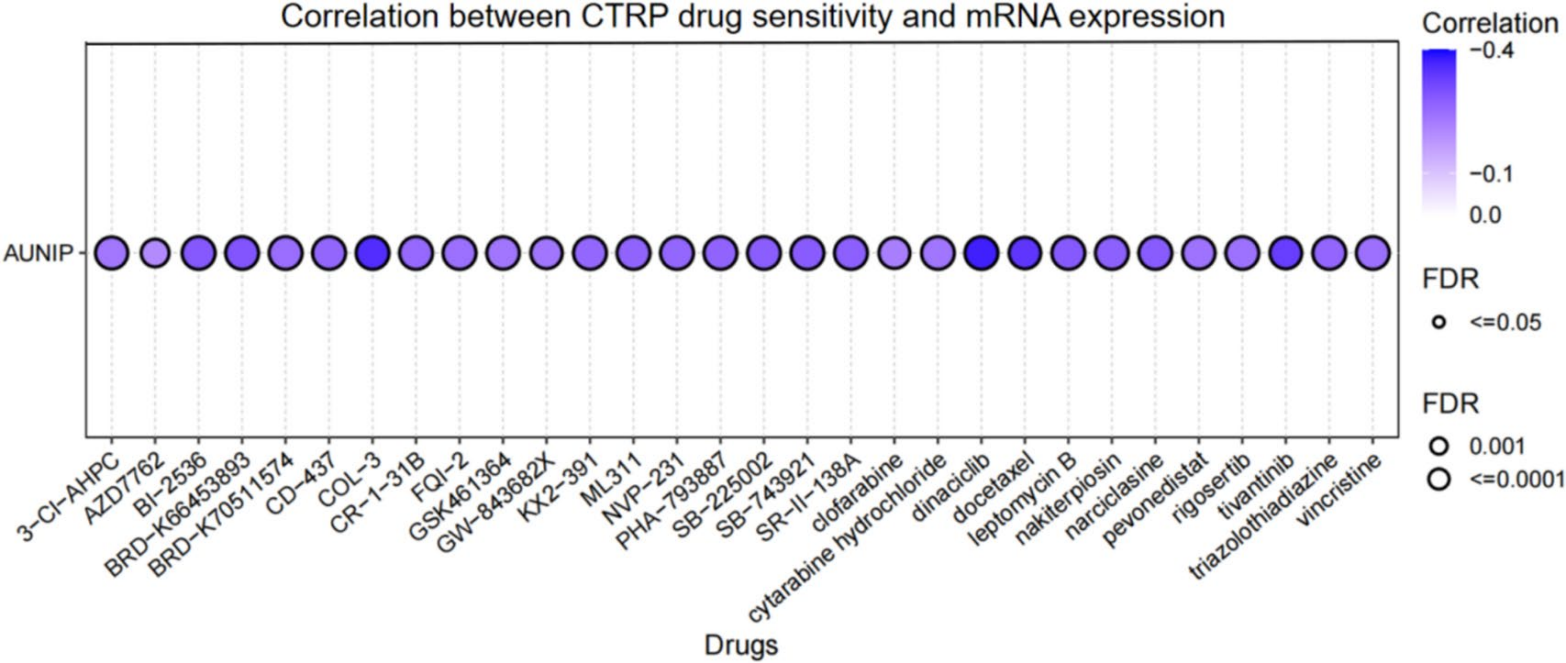
The correlation between AUNIP expression and drug sensitivity using the CTRP dataset

We found that AUNIP expression was negatively linked with 50% inhibitory concentration (IC50) values of 30 drugs based on the results of the CTRP dataset in GSCA. There was a strong negative correlation with IC50 of COL-3, dinaciclib, and docetaxel (Fig. 7). These findings indicated that AUNIP was significantly associated with different drug sensitivities in various tumor cell lines and may be a latent target for cancer therapy.

### Gene functional enrichment of AUNIP in pan-cancer

**Fig. 8:**
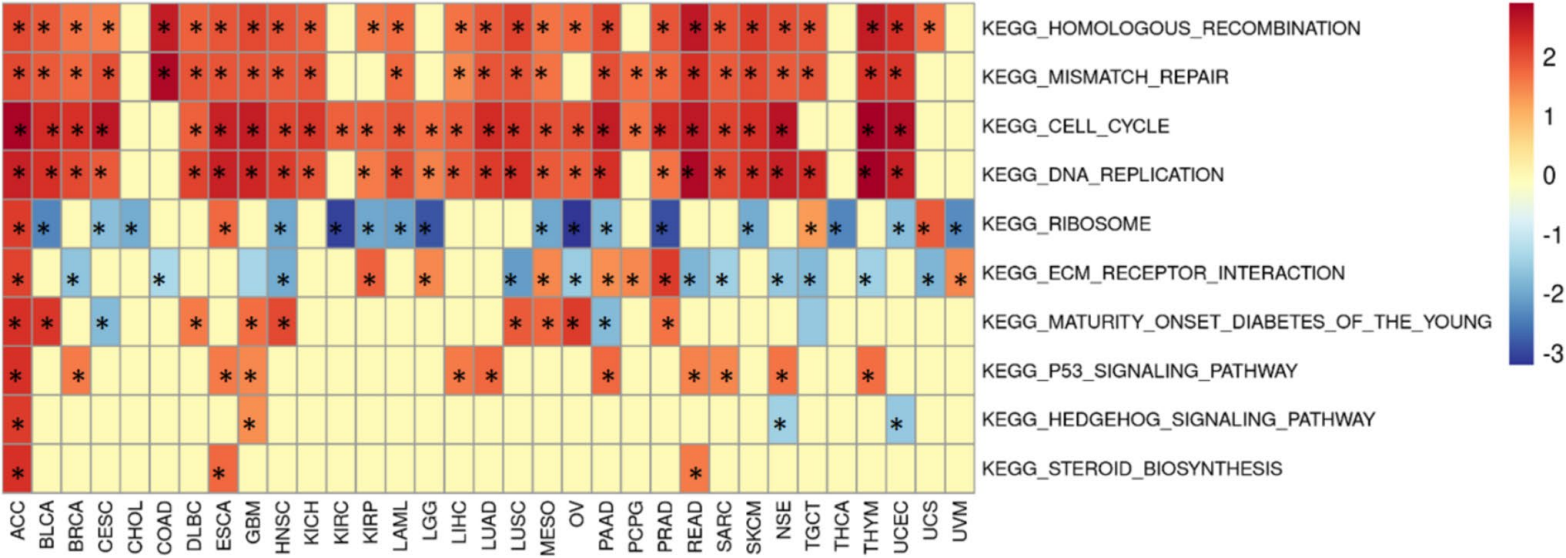
The functional enrichment of AUNIP in pan-cancer using GSEA

The results of GSEA demonstrated that AUNIP was primarily participated in cell cycle, DNA replication, mismatch repair, and homologous recombination in most tumors (Fig. 8). In these tumors, AUNIP was mainly involved in the development of tumors through the above pathways.

### Overexpression AUNIP was correlated with clinical information in LIHC and an independent prognostic gene for LIHC

**Fig. 9:**
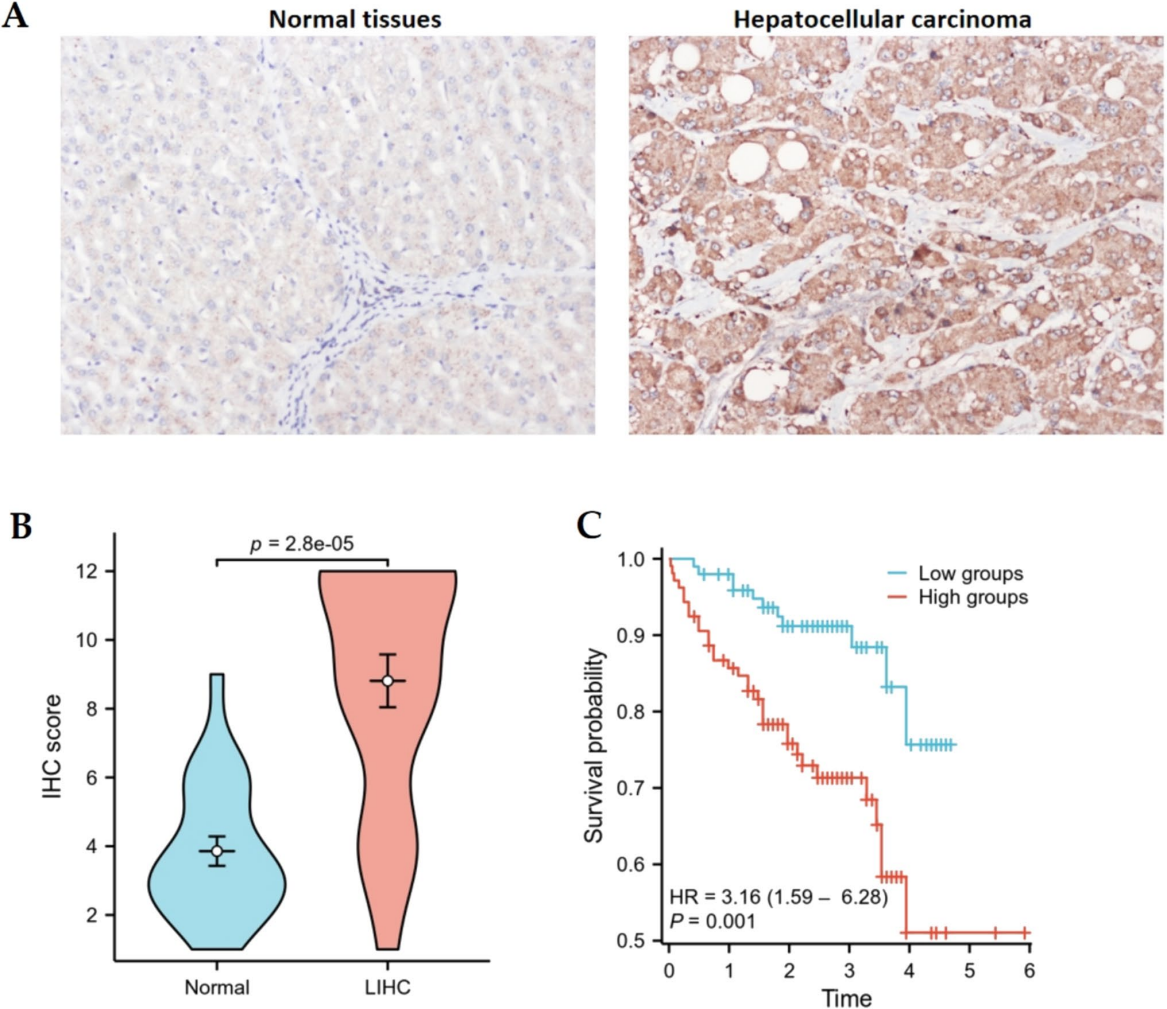
The expression of AUNIP in LIHC (**A**) and Kaplan–Meier analysis of AUNIP in LIHC (**B**)

IHC analysis demonstrated that AUNIP was overexpressed, compared to normal liver tissues (Fig. 9A,B). AUNIP expression was linked with histologic grade, not correlated with age, gender, and pathologic stage (Table 1). Kaplan–Meier analysis suggested that the patients with high-expression group had worse prognosis (Fig. 9C).

**Table 1:** The correlation of AUNIP expression with clinical information in LIHC

Characteristics	AUNIP		*χ*^2^	*P*
	Low expression	High expression		
*Age*			0.25	0.72
≤ 55	14	20		
> 55	5	5		
*Gender*			0.24	0.63
Female	10	15		
Male	9	10		
*Pathologic stage*			0.09	0.76
Stage I + Stage II	9	13		
Stage III + Stage IV	10	12		
*Histologic grade*			4.54	0.03
G1 + G2	13	9		
G3	6	16		

## Discussion

The global mortality burden is predominantly attributed to malignant neoplasms resulting from dysregulated cellular proliferation. Recent decades have witnessed remarkable progress in early disease detection methodologies, encompassing the conventional approaches including radiotherapy, surgical procedures, tailored therapeutic regimens, and chemotherapeutic interventions (Mishra et al. 2023a, 2023b, 2023c). However, cancer remains a major threat to human health. Therefore, it is important to find an effective biomarker to predict the development and prognosis of cancer. DNA double-strand breakage damage is the most severe form of damage, if not repaired in time or abnormal repair occurs, it will lead to a series of changes in the cell genome, directly lead to deactivation of tumor suppressor genes or overexpression of oncogenes, and eventually lead to cell cancer (Burma et al. 2006). The most critical factor affecting the selection of DNA double-strand break repair pathways is the state of the cut end of DNA, and AUNIP is a key factor regulating the state of DNA cleavage ends. AUNIP, a binding protein of protein kinase A and Ninein proteins, also known as AIBP, is a structurally specific DNA-binding protein that is localized to the 135 open-reading framework of chromosome 1. According to reports, it is highly expressed in various tumors (Ma et al. 2020). AUNIP is highly expressed in astrocytoma and other brain tumors, suggesting that AUNIP may play a role as oncogenic genes in the development of brain tumors (Lieu et al. 2010).

Our study analyzed the expression, clinical significance, prognosis, mutation, and immunity of AUNIP from the perspective of pan-cancer using a multi-omics system. It was found that AUNIP expression was increased significantly in most tumors compared to normal tissues, suggesting that AUNIP may be a key gene in cancer development. We conducted IHC analysis to confirm the higher expression of AUNIP in LIHC, which was consistent with TCGA database. In addition, AUNIP with high expression in ACC, LGG, LIHC, MESO, and SARC had poorer OS and DFS than those of AUNIP with low expression, suggesting that the high expression of AUNIP in some tumors influenced patients’ prognosis. Furthermore, AUNIP expression was related to the T stage, N stage, and clinicopathological stage in some cancers, indicating that AUNIP may be a promising valuable diagnostic and prognostic marker in multiple tumors. IHC analysis indicated that AUNIP was linked with histologic grade in LIHC. Moreover, AUNIP expression was an independent prognostic index by univariate and multivariate regression in LIHC. We used cBioportal to study the frequency of AUNIP gene alteration in tumors. In CHOL, the frequency of genetic changes was the highest, with all deep deletions, followed by PCPG, with all deep deletions.

Immune cell infiltration is closely linked to cancer progression (Marcas and Walzer 2018). Recent studies have suggested that tumor progression is caused by an imbalance between the tumor's immune state and the host's immune response (Nabbi et al. 2019). We studied the relationship between AUNIP and immunocyte infiltration and observed that AUNIP expression was positively related to Th2 for most tumors, indicating that with the increase of AUNIP expression, Th2 concentration was up-regulated. Th2 cells are not conducive to the anti-tumor effect of cellular immunity. Th1/Th2 drift will protect the tumor from immune surveillance and immune attack, thus promoting the development and progression of tumors (Sharma et al. 2007). Furthermore, we applied the ESTIMATE algorithm to discuss the correlation between AUNIP and stromalscore, immunescore, and estimatescore in different tumors. In most tumors, AUNIP was negatively correlated with these three scores. The application in immune checkpoint inhibitors has elevated immunotherapy to a new level. Immunotherapy has been considered as an effective therapy for various advanced and invasive cancers (Morse et al. 2005; Zhou and Zhong 2004). At present, immunotherapy has been applied to a variety of tumors. Common immune checkpoints include PD-1, PD-L1, CTLA-4, PDCD1LG2, TIGIT, HAVCR2, SIGLEC15, and LAG3. In some tumors, AUNIP expression was positively related to immune checkpoint expression, suggesting that these patients with high AUNIP expression may benefit from immunotherapy. TMB and MSI are effective markers to predict the effect of immunotherapy. MSI-H's tumor gene repair system is abnormal, and there may be more gene mutations, which are easily recognized by T cells and may respond better to immunotherapy (Bateman 2021). The higher the TMB, the greater the probability that neoantigens expressed by the tumor will be identified by the immune system. Therefore, tumors with high TMB are more sensitive to immune therapy (Liu et al. 2019). Our study demonstrated that AUNIP had positive association with TMB and MSI in BLCA, SARC, and STAD, and patients with high AUNIP expression in these three types of tumors were more susceptible to immunotherapy.

The results of gene enrichment analysis showed that AUNIP caused tumors progression through the cell cycle, DNA replication, mismatch repair, and homologous recombination in most tumors. This is consistent with the literature reports (Lou et al. 2017) In addition, we also performed a correlative analysis between AUNIP and drug sensitivity, and we used a public database to predict several candidate targeted small-molecule drugs. We found that AUNIP was negatively related to IC50 values of 30 drugs, indicating that these drugs stop the progression of the tumor. This provides a novel insight into expanding the therapeutic selection of these targeted small-molecule drugs and developing new drugs specifically targeting AUNIP.

We performed IHC analysis to discuss AUNIP expression in LIHC and the findings demonstrated that AUNIP expression was up-regulated in LIHC. The patients with high-expression group had unfavorable prognosis. These findings suggested that overexpression AUNIP was correlated with the progress of LIHC development and prognosis.

In our work, the expression, prognosis, and characteristics of AUNIP were elucidated by pan-cancer analysis. However, there are some shortcomings in this study. The characteristics of AUNIP were analyzed through bioinformatics and only conducted IHC to verify the overexpression of AUNIP in LIHC. However, there was no biological experiment to verify it. Therefore, in the following studies, it needs more experiment to further validate the mechanism of effect of AUNIP in inducing tumors.

## Electronic supplementary material

Below is the link to the electronic supplementary material.

Supplementary material 1 (DOCX 333 kb)

## Data Availability

The data included in the research report are included in the article. Further inquiries can be made directly to the corresponding author.

## Abbreviations

ACC: Adrenocortical carcinoma
BLCA: Bladder urothelial carcinoma
BRCA: Breast invasive carcinoma
CESC: Cervical squamous cell carcinoma and endocervical adenocarcinoma
CHOL: Cholangio carcinoma
COAD: Colon adenocarcinoma
DLBC: Lymphoid neoplasm diffuse large B-cell lymphoma
ESCA: Esophageal carcinoma
GBM: Glioblastoma multiforme
HNSC: Head and neck squamous cell carcinoma
KICH: Kidney chromophobe
KIRC: Kidney renal clear cell carcinoma
KIRP: Kidney renal papillary cell carcinoma
LAML: Acute myeloid leukemia
LGG: Brain lower grade glioma
LIHC: Liver hepatocellular carcinoma
LUAD: Lung adenocarcinoma
LUSC: Lung squamous cell carcinoma
MESO: Mesothelioma
OV: Ovarian serous cystadenocarcinoma
PAAD: Pancreatic adenocarcinoma
PCPG: Pheochromocytoma and paraganglioma
PRAD: Prostate adenocarcinoma
READ: Rectum adenocarcinoma
SARC: Sarcoma
SKCM: Skin cutaneous melanoma
STAD: Stomach adenocarcinoma
TGCT: Testicular germ cell tumors
THCA: Thyroid carcinoma
THYM: Thymoma
UCEC: Uterine corpus endometrial carcinoma
UCS: Uterine carcinosarcoma
UVM: Uveal melanoma

## References

[CR1] Bateman AC (2021) DNA mismatch repair proteins: scientific update and practical guide[J]. J Clin Pathol 74(4):264–268 33597222 10.1136/jclinpath-2020-207281

[CR2] Burma S, Chen BP, Chen DJ (2006) Role of non-homologous and joining (NHEJ) in maintaining genomic integrity. DNA Repair (Amst) 5:1042–1048 16822724 10.1016/j.dnarep.2006.05.026

[CR3] Cerami E, Gao J, Dogrusoz U, Gross BE, Sumer SO, Aksoy BA, Jacobsen A, Byrne CJ, Heuer ML, Larsson E et al (2012) The cBio cancer genomics portal: an open platform for exploring multidimensional cancer genomics data. Cancer Discov 2:401–404 22588877 10.1158/2159-8290.CD-12-0095 PMC3956037

[CR4] Chan TA, Yarchoan M, Jaffee E et al (2019) Development of tumor mutation burden as an immunotherapy biomarker: utility for the oncology clinic[J]. Ann Oncol 30(1):44–56 30395155 10.1093/annonc/mdy495 PMC6336005

[CR5] Chou CH, Loh JK, Yang MC, Lin CC, Hong MC, Cho CL, et al. AIBp regulates mitotic entry and mitotic spindle assembly by controlling activation of both Aurora-A and Plk1 10.1080/15384101.2015.1066536 PMC4614063 26114227

[CR6] Dudley JC, Le DT et al (2016) Microsatellite instability as a biomarker for PD-1 blockade[J]. Clin Cancer Res 22(4):813–820 26880610 10.1158/1078-0432.CCR-15-1678

[CR7] Li T, Fu J, Zeng Z, Cohen D, Li J, Chen Q, Li B and Shirley Liu X (2020) TIMER2.0 for analysis of tumor-infiltrating immune cells. Nucleic Acids Res 10.1093/nar/gkaa407 PMC7319575 32442275

[CR8] Lieu A, Cheng T, Chou C et al (2010) Functional characterization of AIBp, a novel Aurora-A binding protein in centrosome structure and spindle formation. Int J Oncol 37:429–436 20596670 10.3892/ijo_00000691

[CR9] Liu L, Bai X, Wang J et al (2019) Combination of TMB and CNA stratifies prognostic and predictive responses to immunotherapy across metastatic cancer [J]. Clin Cancer Res 25(24):7413–7423 31515453 10.1158/1078-0432.CCR-19-0558

[CR10] Liu C-J, Hu F-F, Xie G-Y, Miao Y_R, Li X-W, Zeng Y, Guo A-Y (2022) GSCA: an integrated platform for gene set cancer analysis at genomic, pharmacogenomic, and immunogenomic levels. Briefings Bioinform bbac558 10.1093/bib/bbac558 36549921

[CR11] Lou J, Chen H, Han J, He H, Huen MSY, Feng X-H, Liu T, Huang J (2017) AUNIP/C1orf135 directs DNA double-strand breaks towards the homologous recombination repair pathway. Nat Commun 8(1):985 29042561 10.1038/s41467-017-01151-w PMC5645412

[CR12] Ma C, Kang W, Yu L et al (2020) AUNIP expression is correlated with immune infiltration and is a candidate diagnostic and prognostic biomarker for hepatocellular carcinoma and lung adenocarcinoma. Front Oncol 10:Article 590006 33363020 10.3389/fonc.2020.590006 PMC7756081

[CR13] Marcas A, Walzer T (2018) An immunosuppressive pathway for tumor progression [J]. Nat Med 24(3):260 29509752 10.1038/nm.4508

[CR14] Mishra D, Mishra A, Rai SN, Vamanu E, Singh MP (2023a) Identification of prognostic biomarkers for suppressing tumorigenesis and metastasis of hepatocellular carcinoma through transcriptome analysis. Diagnostics 13:965 36900109 10.3390/diagnostics13050965 PMC10001411

[CR15] Mishra D, Mishra A, Rai SN, Vamanu E, Singh MP (2023b) Demystifying the role of prognostic biomarkers in breast cancer through integrated transcriptome and pathway enrichment analyses. Diagnostics 13:1142 36980449 10.3390/diagnostics13061142 PMC10046968

[CR16] Mishra D, Mishra A, Rai S, Singh S, Vamanu E, Singh MP (2023c) In Silico Insight to Identify Potential Inhibitors of BUB1B from Mushroom Bioactive Compounds to Prevent Breast Cancer Metastasis. Front. Biosci. Landmark Ed 28(7):151 37525917 10.31083/j.fbl2807151

[CR17] Morse MA, Garst J, Osada T et al (2005) A phase I study of dexosome immunotherapy in patients with advanced non-small cell lung cancer [J]. J Transl Med 3(1):9 15723705 10.1186/1479-5876-3-9 PMC551593

[CR18] Nabbi A, Jager N, Sudhaman S et al (2019) Landscape of infiltrating immune repertoire in pediatric solid tumors[J]. Proc Natl Acad Sci 116(47):23662–2367031685621

[CR19] Sharma A, Rajappa M, Saxena A et al (2007) Cytokine profile in Indian women with cervical intraepithelial neoplasia and cancer cervix[J]. Int J Gynecol Cancer 4(17):879–885 10.1111/j.1525-1438.2007.00883.x 17343606

[CR20] Tang Z et al (2019) GEPIA2: an enhanced web server for large-scale expression profiling and interactive analysis. Nucleic Acids Res. 10.1093/nar/gkz430 31114875 10.1093/nar/gkz430 PMC6602440

[CR21] Yang L, Yang S, Yuan X et al (2018) Analyzing pan-cancer DNA methylation patterns via clustering[J]. J Xidian Univ 45(4):23–28

[CR22] Yang Z, Liang X, Fu Y et al (2019) Identification of AUNIP as a candidate diagnostic and prognostic biomarker for oral squamous cell carcinoma. EBioMedicine 47:44–57 31409573 10.1016/j.ebiom.2019.08.013 PMC6796785

[CR23] Zhang K, Wang H (2015) Cancer genome atlas pan-cancer analysis project[J]. Zhongguo Fei Ai Za Zhi 18(4):219–223 25936886 10.3779/j.issn.1009-3419.2015.04.02 PMC6000284

[CR24] Zhang C, Li Z, Qi F et al (2019) Exploration of the relationships between tumor mutation burden with immune infiltrates in clear cell renal cell carcinoma[J]. Ann Transl Med 7(22):648 31930049 10.21037/atm.2019.10.84 PMC6944593

[CR25] Zhou J, Zhong Y (2004) Breast cancer immunotherapy [J]. Cell Mol Immunol 1(4):247–255 16225767

