# Supplementary figures and images for: AUNIP was a candidate marker for prognosis and immunology in pan-cancer
Source: 3 Biotech. 2025 May 17;15(6):177. doi: 10.1007/s13205-025-04294-6 (PMC12085456; doi:10.1007/s13205-025-04294-6)

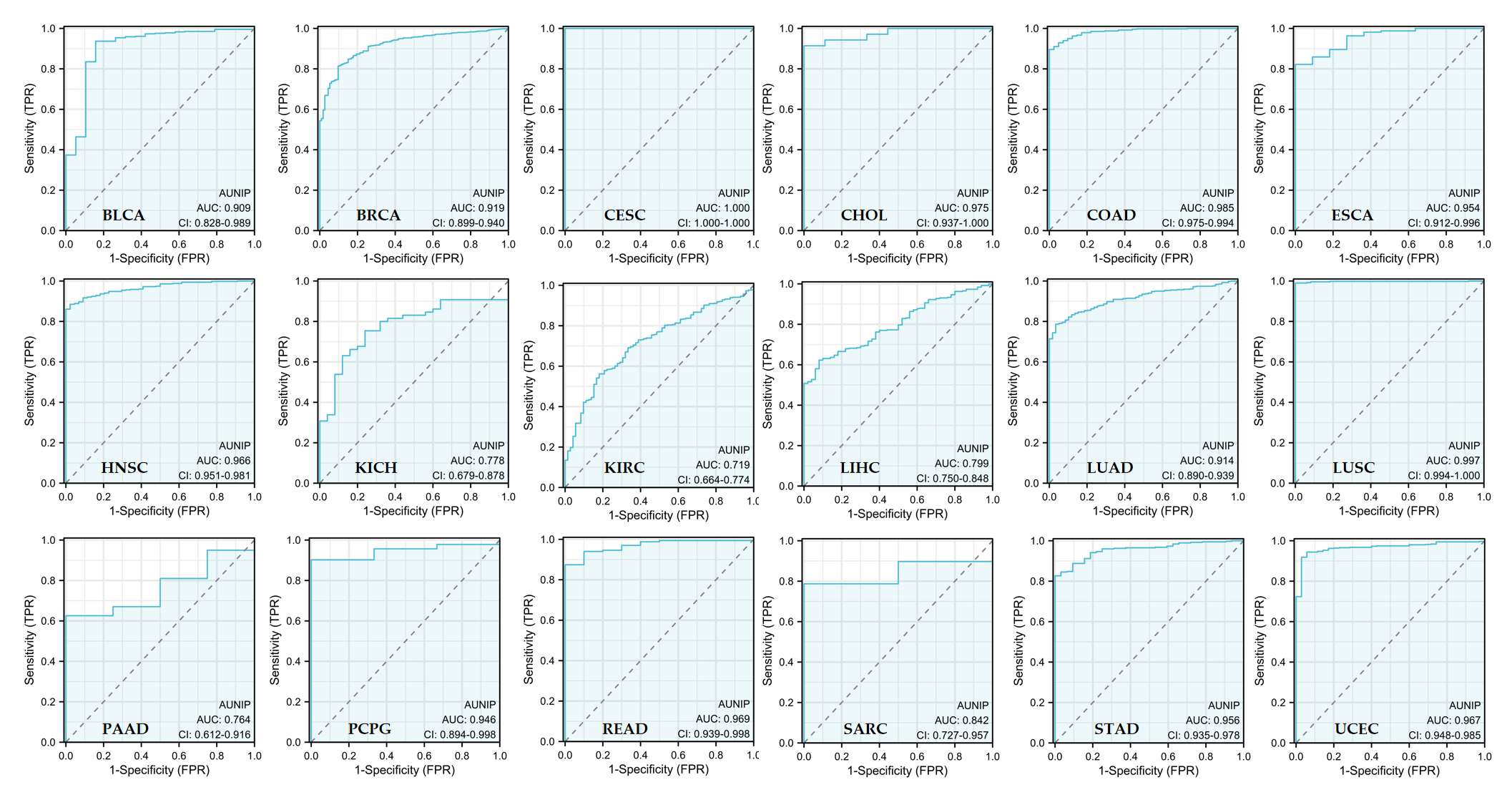


**Figure S1** The diagnostic ROC of AUNIP in pan-cancer

Supplement: Supplementary file 1 — Supplementary material 1 (DOCX 333 kb) [file 13205_2025_4294_MOESM1_ESM.docx]
